# Synthesis of Nanoscale Precipitated Calcium Carbonate: Additive-Free Precipitation and Chain-like Structure Evolution

**DOI:** 10.3390/ma19091879

**Published:** 2026-05-02

**Authors:** Aili Zhou, Xiaolan Song, Xiaoqin Li, Qisen Zhang, Shengming Jin, Kuixin Cui

**Affiliations:** 1 School of Minerals Processing and Bioengineering, Central South University, Changsha 410083, China; ailii_zhou@163.com (A.Z.); songxiaolan@csu.edu.cn (X.S.); 2 School of Environment and Energy, Guangdong Provincial Key Laboratory of Solid Wastes Pollution Control and Recycling, South China University of Technology, Guangzhou 510006, China; 3 Fujian Sanming Zhengyuan Chemical Co., Ltd., Sanming 365508, China

**Keywords:** nano-precipitated calcium carbonate, chain-like structures, additive-free synthesis

## Abstract

Nanoscale precipitated calcium carbonate (NPCC) is a versatile functional material whose performance is highly governed by particle size, morphology, and dispersion state. Conventional synthesis methods often rely on complex additives or multi-step processing, thereby impeding scalability and precise morphological regulation. Herein, we present a simple and additive-free route for the tunable synthesis of NPCC via the direct reaction of an aqueous Na_2_CO_3_ solution with a Ca(OH)_2_ suspension under ambient conditions. A systematic investigation was conducted to elucidate the influence of key synthetic parameters—namely, reactant concentration, temperature, injection rate, stirring speed, and aging duration—on the resultant particle characteristics. Under optimal conditions, cubic NPCC with a mean particle size of approximately 90 nm, distinct crystalline features, and good dispersion was successfully obtained. Furthermore, integrated analysis employing time-resolved pH and conductivity monitoring, electron microscopy, and diffraction techniques revealed a growth trajectory distinct from the classical crystallization pathway of CaCO_3_. This observed behavior suggests a mechanistic association with non-classical crystallization. These findings not only furnish a practical and environmentally benign strategy for the production of high-quality NPCC but also offer fundamental insights into the crystallization mechanisms of calcium carbonate, with broader implications for the rational design of advanced inorganic nanomaterials.

## 1. Introduction

The synthesis of functional inorganic nanomaterials has become a central focus of modern materials science, driven by their unique properties and broad applicability across advanced industrial sectors [1,2,3,4]. Among these materials, nanoscale precipitated calcium carbonate [5] (NPCC) is of particular interest due to its excellent biocompatibility, high specific surface area, tunable morphology, and widespread use in composites, coatings, pharmaceuticals, and environmental technologies [6,7,8,9]. The performance of NPCC in these applications is intrinsically linked to its particle size, dispersion state, and crystalline morphology, thereby establishing the development of reliable and scalable synthesis routes as a critical research objective [10,11,12,13]. More broadly, recent critical analyses of oxide-based nanomaterial systems have emphasized that material performance cannot be understood solely in terms of nominal composition but must also be interpreted in relation to synthesis-controlled structural and interfacial parameters, which ultimately govern structure–property–performance relationships [14].

Conventional methods for producing NPCC, such as carbonation [15,16], double decomposition [17], emulsion [18,19], and sol–gel processes [20,21], often rely on complex additives, multi-step procedures, or stringent reaction conditions [22,23,24]. For instance, Sun et al. [25] synthesized nano-CaCO_3_ by simultaneously absorbing CO_2_ and NH_3_ into a CaCl_2_ solution within a rotating packed bed (RPB), without the use of any additives. Process parameters, including gas and liquid flow rates, were optimized to control particle size, yielding nano-CaCO_3_ particles with an average size of 50 nm and a narrow size distribution (10–80 nm). Yang et al. [17] synthesized nano-CaCO_3_ by mixing CaCl_2_ and Na_2_CO_3_ solutions with Ca(OH)_2_ added as an additive, observing that an increase in reaction temperature from 10 °C to 30 °C enlarged the average particle size from 50 nm to 110 nm. Utilizing an emulsion method with anhydrous Na_2_CO_3_, kerosene, and Ca(NO_3_)_2_, Sun et al. [26] successfully prepared nano-CaCO_3_ with average particle sizes ranging from 80 to 100 nm. Hussein et al. [27] synthesized spherical CaCO_3_ nanoparticles from cockle shells via a sol–gel method. Optimized conditions involved the addition of 50 mL of double-distilled water to a CaCl_2_ solution in ethanol, followed by the addition of K_2_CO_3_ at a controlled feeding rate over one hour. The resulting nanoparticles exhibited an average size of 39 nm and a surface area of 26 m^2^/g. These approaches, however, can introduce impurities, increase production costs, and limit the uniformity and reproducibility of the final product [28,29,30,31]. Although significant progress has been made in obtaining nanoscale CaCO_3_, precise control over particle morphology and dispersion without resorting to organic modifiers or complex equipment remains a considerable challenge. A particular gap exists in understanding the dynamic crystallization pathways and transient structural evolution, such as the formation and disintegration of intermediate chain-like assemblies, which are crucial for achieving tailored material properties.

In contrast to previous studies, this work reports a simple, additive-free, and readily controllable precipitation route for synthesizing high-quality NPCC using Na_2_CO_3_ solution and Ca(OH)_2_ suspension as raw materials under mild ambient conditions. By systematically regulating key parameters, including reactant concentration, temperature, injection rate, stirring intensity, and aging time, we demonstrate that the particle size, morphology, and dispersibility of the resulting CaCO_3_ can be effectively modulated under additive-free conditions. More importantly, in-process monitoring combined with ex situ characterization provided insight into the dynamic evolution of transient chain-like superstructures during crystallization, including their formation and subsequent fragmentation. This work not only provides a practical and environmentally benign strategy for producing NPCC with tunable particle characteristics but also offers fundamental insights into the non-classical crystallization pathways of calcium carbonate, with potential implications for the design of other functional nanomaterials.

## 2. Materials and Methods

### 2.1. Materials

Sodium carbonate (Na_2_CO_3_) and calcium hydroxide (Ca(OH)_2_) of analytical grade were purchased from Sinopharm Chemical Reagent Co., Ltd. (Shanghai, China). All chemicals were used as received without further purification. Deionized water was utilized for the preparation of Na_2_CO_3_ solutions and Ca(OH)_2_ suspensions.

### 2.2. Synthesis of Nanoscale Precipitated Calcium Carbonate

Nanoscale precipitated calcium carbonate (NPCC) was synthesized via a precipitation reaction between Na_2_CO_3_ and Ca(OH)_2_ suspension in a 500 mL double-walled glass reactor equipped with a constant-temperature water bath. A 100 mL Ca(OH)_2_ suspension was introduced into the reactor and maintained at 15–35 °C under stirring at 200–1000 rpm. Subsequently, 100 mL of Na_2_CO_3_ solution was continuously pumped into the Ca(OH)_2_ suspension at an injection rate of 5–200 mL/min using a peristaltic pump. The concentrations of the Ca(OH)_2_ suspension and Na_2_CO_3_ solution were varied in the range of 0.04–0.20 mol/L to examine their effects on NPCC formation. After complete addition of the Na_2_CO_3_ solution, the suspension was aged for 0.5–8 h under continuous stirring. After completion of the reaction, the resulting precipitate was subjected to vacuum filtration and washed sequentially three times with deionized water and anhydrous ethanol. The filtered CaCO_3_ powder was transferred onto a watch glass and dried at 60 °C for 12 h. To prevent moisture absorption, the dried product was promptly sealed, labeled, and stored in a desiccator for subsequent analysis.

### 2.3. Characterization

The pH variation throughout the precipitation process was continually recorded using a pH meter (pHFE28, Mettler Toledo, Greifensee, Switzerland). Simultaneously, the conductivity of the reaction system was monitored in real time with a conductivity meter (DDSJ-308F, Shanghai Leici Instruments Co., Ltd., Shanghai, China). The morphology and particle size of the as-synthesized CaCO_3_ were examined by scanning electron microscopy (SEM; TESCAN MIRA4,TESCAN, Brno, Czech Republic) and high-resolution transmission electron microscopy (HR-TEM; Tecnai G2 60-300, FEI, Hillsboro, OR, USA). Crystalline structure and phase identification were performed by X-ray diffraction (XRD; Rigaku Smart Lab, Rigaku, Tokyo, Japan) with Cu Kα radiation at a scanning speed of 5°/min. The particle size of NPCC was further determined statistically using ImageJ software (version 1.54p) based on electron micrographs. The particle size distribution of the agglomerated samples was determined by laser diffraction analysis using a BT-9300 ST instrument (Bettersize Instruments Ltd., Shenzhen, China).

## 3. Results and Discussion

### 3.1. Influence of Reaction Conditions on the Synthesis of NPCC

The precipitation of CaCO_3_ encompasses several stages, including reactant mixing, chemical reaction, nucleation, and crystal growth. Understanding how various reaction parameters influence the particle size, morphology, and crystal structure of synthetic CaCO_3_ is essential for tailoring specific crystalline phases and morphologies to enhance performance in targeted applications. In this work, the effects of key synthesis parameters, namely, initial reactant concentrations (0.04–0.2 mol/L), reaction temperatures (15–35 °C), injection rate of Na_2_CO_3_ solution (5–200 mL/min), stirring speed (200–1000 rpm), and aging time (0.5–8 h), on the particle size and morphology of CaCO_3_ were systematically investigated.

#### 3.1.1. Effect of Initial Reactant Concentration on CaCO_3_

The precipitation behavior of CaCO_3_ is strongly influenced by the initial reactant concentration, which governs the supersaturation level of the system and consequently affects reaction kinetics, nucleation behavior, crystal growth, and particle size distribution. Therefore, careful regulation of reactant concentration is important to obtain CaCO_3_ with a desirable particle size and dispersibility. The present reaction system involves the simultaneous dissolution of Ca(OH)_2_, precipitation of CaCO_3_, and formation of NaOH, creating a highly dynamic environment. As such, supersaturation must be carefully regulated to prevent excessive nucleation or particle aggregation. To investigate the effect of reactant concentration on NPCC preparation, we conducted the experiments under the following conditions: a reaction temperature of 25 °C, a Na_2_CO_3_ solution injection rate of 5 mL/min, a stirring speed of 600 rpm, and an aging time of 0.5 h.

**Figure 1:**
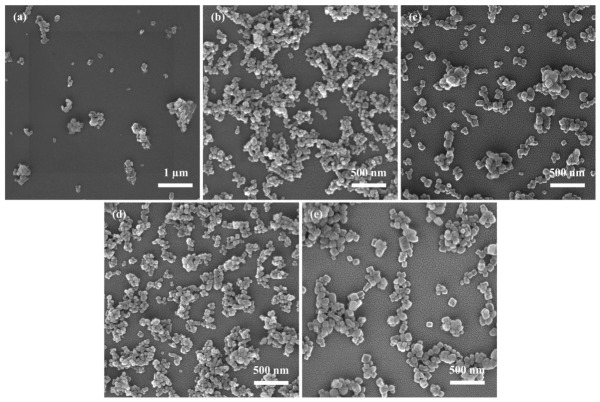
SEM images of CaCO_3_ under different initial reactant concentrations: (**a**) 0.04 mol/L; (**b**) 0.08 mol/L; (**c**) 0.12 mol/L; (**d**) 0.16 mol/L; (**e**) 0.20 mol/L.

Figure 1a–e illustrate the morphological evolution of the synthesized NPCC under different initial reactant concentrations. At 0.04 mol/L (Figure 1a), CaCO_3_ primarily forms aggregates with a D_50_ of 2.7 μm. A limited number of nanoscale particles are observed, exhibiting poor uniformity and sparse distribution. This can be attributed to the low supersaturation under dilute conditions, which leads to a slow reaction rate and limited nucleation. Under such conditions, fewer nucleation sites are available, promoting continuous crystal growth and the formation of large particles or aggregates. Additionally, the high surface energy of fine particles may contribute to particle agglomeration. When the concentration is increased to 0.08 mol/L (Figure 1b), the aggregation is markedly reduced. The resulting CaCO_3_ particles are uniformly distributed with sizes below 100 nm. This improvement stems from the increased supersaturation, which enhances the nucleation rate and generates a large number of nuclei in the early reaction stage, thereby suppressing uncontrolled crystal growth. Moreover, improved interparticle interactions contribute to a more dispersed morphology. At 0.12 mol/L (Figure 1c), the particles still retain a predominantly fine morphology, but local aggregation begins to appear compared with the sample prepared at 0.08 mol/L. Upon further increasing the concentration to 0.16 mol/L (Figure 1d), the aggregation becomes more evident, and the particle distribution becomes less homogeneous. These intermediate morphologies suggest a gradual transition from the well-dispersed state obtained at 0.08 mol/L toward a less uniform structure at higher concentrations. However, at a higher concentration of 0.2 mol/L (Figure 1e), the increased concentrations of Ca^2+^ and CO_3_^2−^ ions lead to a condition where crystal growth outpaces nucleation, resulting in significant particle aggregation and reduced uniformity.

In summary, an appropriate increase in reactant concentration promotes nucleation and helps suppress excessive particle growth and aggregation, yielding more uniform and finer CaCO_3_ particles. However, both excessively low and high concentrations adversely affect crystal size control and dispersibility. Based on these observations, a reactant concentration of 0.08 mol/L was selected for subsequent experiments.

#### 3.1.2. Effect of Reaction Temperature on CaCO_3_

Temperature plays a critical role in governing the crystalline phase of CaCO_3_. Variations in reaction kinetics and crystal growth mechanisms under different thermal conditions can significantly influence the selectivity and transformation pathway among polymorphs. Low temperatures generally favor the formation of vaterite or aragonite, whereas high temperatures promote the crystallization and structural rearrangement of calcite. In addition, temperature modulates the diffusion rate of ions in solution, thereby affecting the final particle size and morphology of the product. Therefore, a systematic investigation of the temperature-dependent behavior of CaCO_3_ is the directed control of its crystal form and functional performance. To investigate the effect of reaction temperature on NPCC preparation, experiments were carried out under the following conditions: a reactant concentration of 0.08 mol/L, a Na_2_CO_3_ solution injection rate of 5 mL/min, a stirring speed of 600 rpm, and an aging time of 0.5 h.

**Figure 2:**
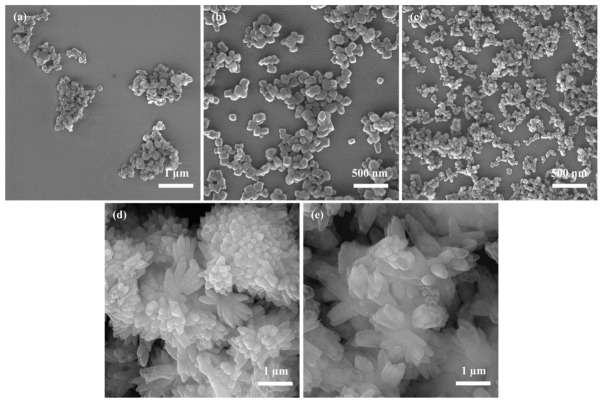
SEM images of CaCO_3_ synthesized under different reaction temperatures: (**a**) 15 °C; (**b**) 20 °C; (**c**) 25 °C; (**d**) 30 °C; (**e**) 35 °C.

Figure 2a–e present SEM images of CaCO_3_ synthesized at different reaction temperatures (15, 20, 25, 30, and 35 °C), illustrating the pronounced influence of temperature on particle morphology. At 15 °C, as shown in Figure 2a, CaCO_3_ primarily consists of aggregates composed of small nanoparticles. This may be attributed to the fact that, at lower reaction temperatures, the reduced ion diffusion and crystal growth rates favor the formation of numerous nuclei. However, these nuclei cannot grow and rearrange sufficiently to form well-dispersed crystals, and their high surface energy makes them prone to aggregation, thereby leading to the aggregation of CaCO_3_ particles. At moderate temperatures of 20 °C and 25 °C, as shown in Figure 2b,c, a notable improvement in particle dispersion and uniformity is observed. The samples synthesized at 25 °C exhibit the most uniform and finest particles with optimal dispersibility, indicating that this temperature favors a balanced kinetics between nucleation and growth. Such conditions help maintain a high nucleation rate while minimizing agglomeration, thereby facilitating the formation of well-defined NPCC. With a further increase in temperature (30 °C and 35 °C, Figure 2d,e), the CaCO_3_ particles begin to form irregular aggregates and develop complex radial clusters, losing their well-defined cubic morphology and exhibiting increased surface roughness. This morphological degradation is likely associated with the change in crystal growth dynamics at elevated temperatures. Higher temperatures accelerate ion diffusion and surface growth, which can disturb the kinetic balance required to maintain a uniform cubic morphology. As a result, crystal growth becomes less controlled, and different crystal faces may develop at unequal rates, leading to surface roughening and irregular particle shapes. Meanwhile, enhanced Brownian motion increases the collision probability among particles, favoring particle attachment and aggregation. Consequently, the products obtained at higher temperatures tend to evolve into irregular aggregates and radial clusters rather than well-dispersed cubic nanoparticles. Since the CaCO_3_ obtained at 30 °C already exhibits significant irregularity, temperatures above 35 °C were not investigated in this study.

Higher temperatures generally introduce disorder into crystal growth, often resulting in particles lacking well-defined facets and exhibiting complex surface structures. Moreover, in the absence of crystal modifiers, the accelerated growth rate at elevated temperatures leads to anisotropic development of crystal faces, rendering facet regulation unfeasible and ultimately yielding CaCO_3_ with rough surfaces and irregular shapes.

In summary, low temperatures (15 °C) promote aggregation due to excessive nucleation and restricted growth; moderate temperatures (20–25 °C) enable the formation of uniformly dispersed NPCC; and higher temperatures (≥30 °C) induce aggregation and disordered growth, resulting in complex and irregular aggregates. Based on these findings, a reaction temperature of 25 °C was selected for subsequent experiments.

#### 3.1.3. Effect of Na_2_CO_3_ Solution Injection Rate on CaCO_3_

In this experiment, the chemical reaction was initiated by injecting the Na_2_CO_3_ solution into the Ca(OH)_2_ suspension. The concurrent processes of Ca(OH)_2_ dissolution, CaCO_3_ precipitation, and NaOH formation result in a highly dynamic system, necessitating precise control over solution supersaturation to prevent excessive nucleation or particle aggregation. The injection rate of the Na_2_CO_3_ solution governs the local rate of change in ion concentration, thereby directly influencing supersaturation, nucleation behavior, and crystal aggregation during the reaction. We conducted the experiments on the effect of the Na_2_CO_3_ solution injection rate on NPCC preparation under the following conditions: a reactant concentration of 0.08 mol/L, a reaction temperature of 25 °C, a stirring speed of 600 rpm, and an aging time of 0.5 h.

**Figure 3:**
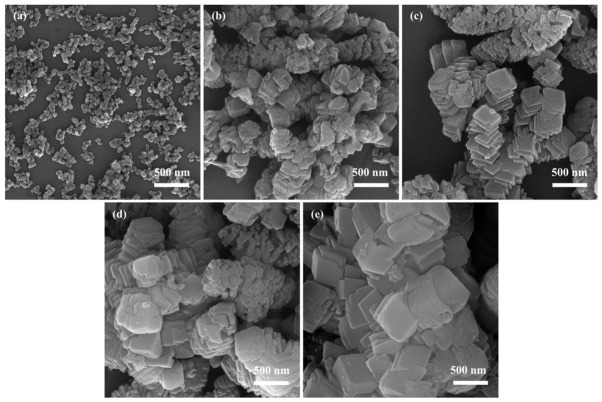
SEM images of CaCO_3_ synthesized under different Na_2_CO_3_ solution injection rates: (**a**) 5 mL/min; (**b**) 50 mL/min; (**c**) 100 mL/min; (**d**) 150 mL/min; (**e**) 200 mL/min.

Figure 3a–e illustrate the morphological evolution of CaCO_3_ under different Na_2_CO_3_ injection rates. At 5 mL/min (Figure 3a), the obtained CaCO_3_ particles exhibit small size, smooth surfaces, a regular morphology, and a uniform size distribution. The slow injection rate is beneficial for the synthesis of NPCC because it allows the reaction to proceed in a near-stoichiometric manner, resulting in a gradual increase in CO_3_^2−^ concentration. This suppresses excessive nucleation and uncontrolled crystal growth, thereby favoring the formation of uniform nanoparticles. When the Na_2_CO_3_ solution was injected at a rate of 5 mL/min, the relatively slow injection rate may have allowed the nucleation and growth rates of CaCO_3_ crystals in the solution to remain more balanced, thereby facilitating the formation of NPCC. In contrast, at higher injection rates (>5 mL/min, Figure 3b–e), the CaCO_3_ crystals appear as agglomerates with stacked lamellar-like structures. Rapid injection leads to inadequate mixing and a sharp local increase in CO_3_^2−^ concentration, causing the system to rapidly exceed the instantaneous supersaturation threshold. This triggers synchronous nucleation and rapid crystal growth, resulting in competitive growth among crystals. As the reaction rate surpasses the ion diffusion rate, disordered crystal stacking and pronounced face-preferred orientation occur, ultimately yielding complex and non-uniform morphologies. These effects stem from severe fluctuations in Ca^2+^ and CO_3_^2−^ concentrations, which induce heterogeneous nucleation and rapid surface growth. Such conditions impede the self-limiting regulation of particles, lead to loss of crystal growth control, and exacerbate aggregation and coarsening of CaCO_3_.

In summary, the injection rate of Na_2_CO_3_ solution significantly influences the particle size, morphology, and structure of CaCO_3_. A low injection rate of Na_2_CO_3_ solution promotes the formation of regularly shaped CaCO_3_, whereas excessively high rates cause rapid mixing of Ca^2+^ and CO_3_^2−^ ions, leaving insufficient time for oriented arrangement into ordered crystals. This results in the formation of a typical block-like interlocking structure. Therefore, an injection rate of 5 mL/min was selected for subsequent experiments.

#### 3.1.4. Effect of Stirring Rate on CaCO_3_

The experiments were performed under the following conditions: a reactant concentration of 0.08 mol/L, a reaction temperature of 25 °C, a Na_2_CO_3_ solution injection rate of 5 mL/min, and an aging time of 0.5 h.

**Figure 4:**
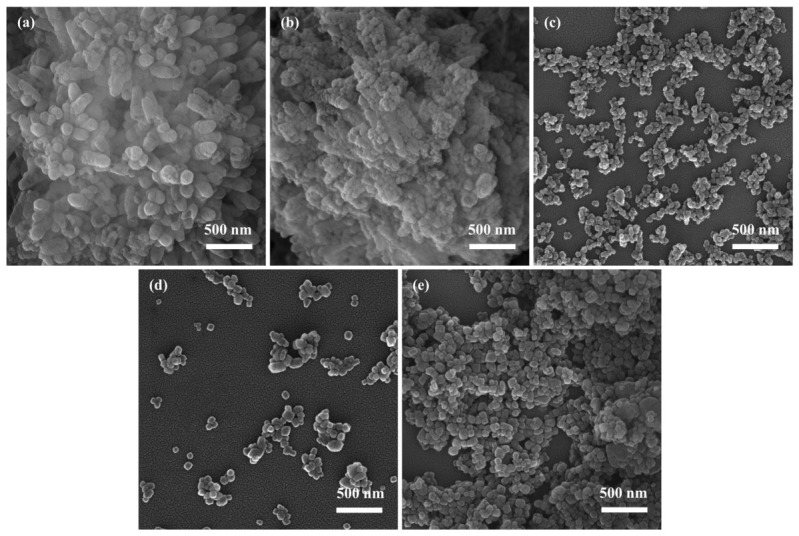
SEM images of CaCO_3_ under different stirring rates: (**a**) 200 rpm; (**b**) 400 rpm; (**c**) 600 rpm; (**d**) 800 rpm; (**e**) 1000 rpm.

Figure 4a–e present the morphological evolution of CaCO_3_ under different stirring rates. At low stirring rates (200–400 rpm, Figure 4a,b), the CaCO_3_ particles exhibit highly irregular shapes and severe aggregation. This can be attributed to insufficient mass transfer under weak agitation, which leads to localized reaction zones and prolonged reaction times, thereby favoring uncontrolled particle agglomeration. When the stirring rate is increased to 600 rpm, as shown in Figure 4c, the CaCO_3_ crystals exhibit a well-defined cubic morphology with improved uniformity. This result is likely due to the more homogeneous mixing conditions under moderate stirring, which improve the distribution of reactants and reduce local concentration gradients in the reaction medium. Such a relatively stable crystallization environment is favorable for more uniform crystal growth and helps suppress uncontrolled aggregation, thereby promoting the formation of well-defined cubic particles. However, further increasing the stirring rate to 1000 rpm (Figure 4e) results in a marked decline in particle uniformity and increased agglomeration. Under such intense agitation, the high mechanical shear, limited by the geometry of the reaction vessel, induces excessive bubble formation. These bubbles interfere with the reaction between CO_3_^2−^ and Ca^2+^ ions, disrupt the crystallization environment, and ultimately lead to a broader particle size distribution and structural irregularity.

Based on the aforementioned analysis, the stirring rate plays an important role in regulating the morphology and particle size of CaCO_3_. Insufficient stirring may lead to uneven reactant diffusion and promote aggregation, whereas excessively high stirring may introduce bubble-related disturbances that affect uniformity. Since the SEM results showed no significant difference in the morphology and particle size of NPCC prepared at 600 rpm and 800 rpm, 600 rpm was selected as a suitable stirring condition for the subsequent experiments because it could provide comparable NPCC formation while operating at a lower stirring intensity.

#### 3.1.5. Effect of Aging Time on CaCO_3_

The influence of aging time on NPCC preparation was examined under the following conditions: a reaction concentration of 0.08 mol/L, a reaction temperature of 25 °C, a Na_2_CO_3_ solution injection rate of 5 mL/min, and a stirring speed of 600 rpm.

**Figure 5:**
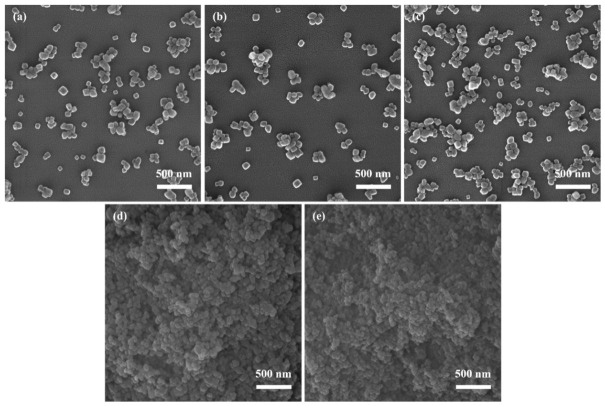
SEM images of CaCO_3_ under different aging times: (**a**) 0.5 h; (**b**) 1 h; (**c**) 2 h; (**d**) 4 h; (**e**) 8 h.

Figure 5a–e present the SEM images of CaCO_3_ obtained under different aging times. As shown in Figure 5a–c, an appropriate aging duration promotes crystal growth, enabling the formation of well-developed CaCO_3_ structures. However, as observed in Figure 5d,e, excessive aging results in pronounced particle aggregation. As summarized in Table 1, the crystallite size of CaCO_3_ was determined through X-ray diffraction (XRD) data analysis and calculated using the Scherrer equation [32]. The inclusion of crystallite size is important for interpreting the crystallization behavior of CaCO_3_ during aging, as it reflects the coherent diffraction domain size and provides information complementary to the particle size observed by SEM and TEM. While electron microscopy mainly reveals the external particle size and aggregation state, crystallite size helps evaluate the evolution of internal crystal domains. The XRD patterns of CaCO_3_ remain essentially unchanged at all aging times, suggesting that the crystal phase is largely stable during aging. In contrast, the crystallite size calculated from XRD data decreases from 78.7 nm at 0.5 h to 49.6 nm at 8 h. This phenomenon may be attributed to the fact that the particles formed at the early stage are metastable polycrystalline CaCO_3_ aggregates, which gradually undergo structural adjustment toward a more stable form during aging. Meanwhile, the decrease in crystallite size is accompanied by an increase in surface energy, thereby favoring the rapid and spontaneous aggregation of the smaller particles. Based on these findings, an aging time of 0.5 h was selected as optimal, as it yields NPCC with regular morphology and good dispersibility while maintaining a stable crystal phase (Figure 5a).

**Figure 6:**
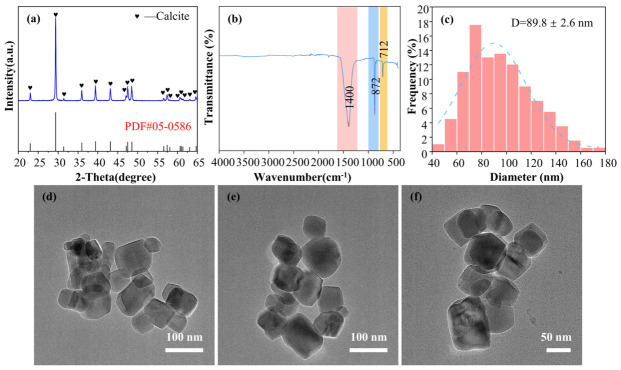
XRD pattern (**a**), FTIR spectrum (**b**), and particle size distribution (**c**) of the as-prepared CaCO_3_; NPCC synthesized under optimized conditions (**d**–**f**).

Figure 6a shows the XRD pattern of the as-prepared NPCC under the optimal condition (0.08 mol/L of Ca(OH)_2_, 25 °C, 5 mL/min of Na_2_CO_3_ solution injection rate, 600 rpm, and aging 0.5 h). The diffraction peaks exhibit sharp profiles, good symmetry, and high intensity, matching well with the standard pattern of calcite (PDF#05-0586). Characteristic diffraction peaks at 2θ values of approximately 23.02°, 29.40°, 35.96°, 39.40°, 43.14°, 47.12°, 47.48°, 48.51°, and 57.4° correspond to the (012), (104), (110), (113), (202), (024), (018), (116), and (122) crystal planes of calcite, respectively. The absence of peaks corresponding to other CaCO_3_ polymorphs or impurities indicates the high phase purity of the obtained calcite. The FT-IR spectrum of CaCO_3_ in Figure 6b further supports the calcite structure, showing characteristic absorption peaks at 712 cm^−1^ (in-plane bending vibration) and 872 cm^−1^ (out-of-plane bending vibration). Particle size distribution analysis using ImageJ software (Figure 6c) indicates an average particle size of D = 89.8 ± 2.6 nm for the as-synthesized CaCO_3_.

The crystallite size of the sample was calculated based on the broadening of the diffraction peaks using the Scherrer equation, as follows:

$$D\;=\;\frac{K\lambda }{\beta \cos\theta }$$

where D is the crystallite size (nm), K is the Scherrer constant, λ is the X-ray wavelength, β is the full width at half maximum (FWHM) of the diffraction peak (rad), and θ is the Bragg diffraction angle. In this work, K = 0.89, λ = 0.15406 nm.

### 3.2. Time-Resolved Evolution of NPCC Precipitation

To investigate the precipitation process between the Ca(OH)_2_ suspension and Na_2_CO_3_ solution, time-resolved pH and conductivity measurements were carried out throughout the reaction. Correspondingly, the morphological evolution and phase composition of the precipitates were characterized at selected intervals using scanning electron microscopy and X-ray diffraction.

#### 3.2.1. Morphological and Phase Evolution of Precipitates

**Figure 7:**
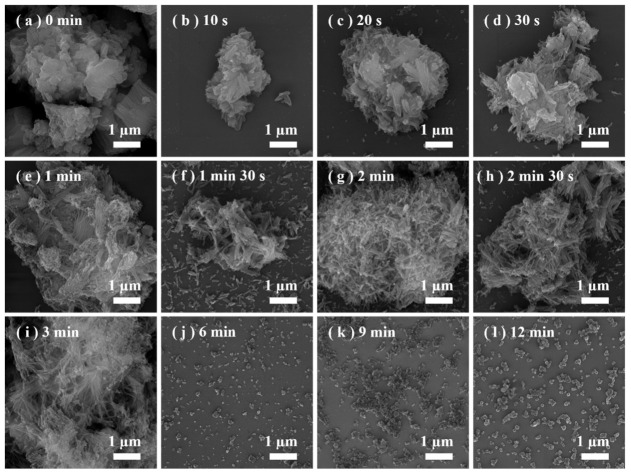
(**a**–**l**) SEM images showing morphological changes during CaCO_3_ growth.

**Figure 8:**
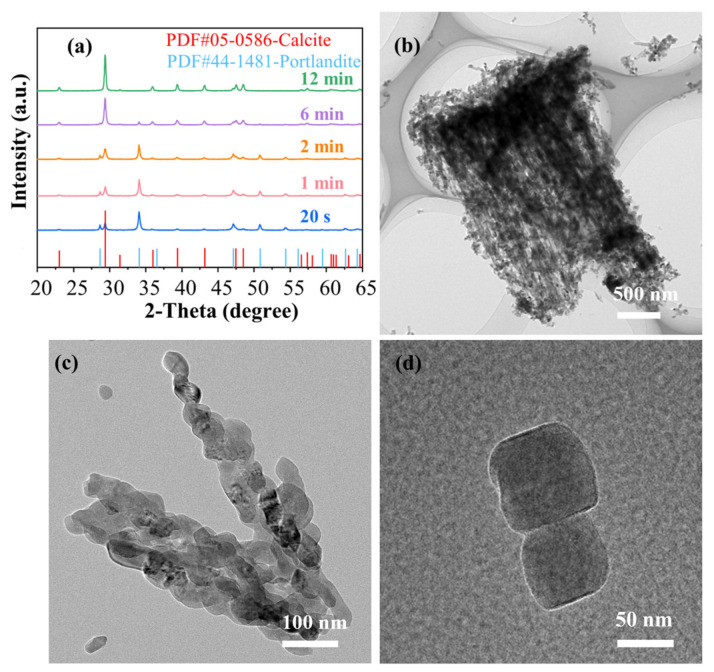
XRD images at different reaction times during the precipitation process (**a**), TEM images of chain-like CaCO_3_ structures (**b**,**c**), and TEM image of CaCO_3_ (**d**).

Samples were collected at different time points during the injection of the Na_2_CO_3_ solution into the Ca(OH)_2_ suspension. The sampled suspensions were then filtered, washed, and dried to obtain powdered samples. These samples obtained at different reaction times were subsequently subjected to SEM and XRD characterization to observe the morphological and phase evolution throughout the reaction process, as shown in Figure 7 and Figure 8.

As shown in Figure 7, the SEM image sequence provides direct evidence of the dynamic morphological evolution during the precipitation process. At the very beginning (0 min, Figure 7a), the suspension consists primarily of irregular Ca(OH)_2_ particles. After adding the Na_2_CO_3_ solution, the nucleation and assembly process initiates rapidly within the first 30 s. Short, chain-like structures composed of nanoparticles begin to emerge (Figure 7b–d). Given that the diffraction peaks shown in Figure 7a belong to CaCO_3_ and Ca(OH)_2_, the newly formed nanoparticles are calcites. These nascent chains elongate and become more pronounced between 1 and 3 min (Figure 7e–i), showing a more developed chain-like transient structure at this stage. Previous studies have reported oriented attachment behavior in CaCO_3_ nanocrystals under aqueous conditions, which provides a reasonable reference for this discussion. Accordingly, the observed linear assemblies may be indicative of an oriented attachment-like process rather than simple random aggregation. Subsequently, a structural disintegration process dominates. From 6 min (Figure 7j) onwards, the continuous networks fragment into shorter segments; accordingly, the peak intensity of Ca(OH)_2_ becomes very weak. This breakdown progresses, and by 12 min (Figure 7l), the morphology transitions to predominantly isolated, individual nanoparticles, and only calcite peaks were detected by XRD (Figure 8), indicating the complete fracture of the metastable chain-like superstructures to well-defined calcites.

#### 3.2.2. Solution Chemistry Monitored by pH and Conductivity

**Figure 9:**
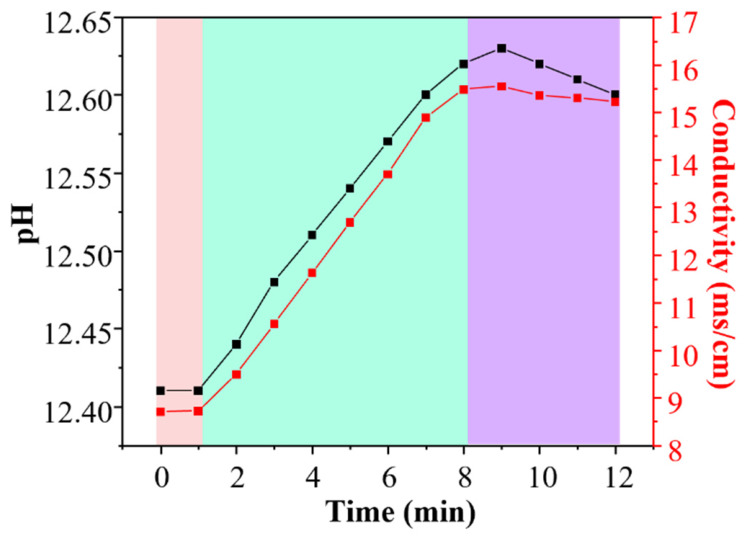
Temporal variations in pH and conductivity throughout the reaction process.

Figure 9 illustrates the temporal variations in pH and conductivity during this transformation and the TEM image of the final product.

As shown in Figure 9, the solution chemistry during this transformation, monitored by pH and conductivity, reveals three distinct regimes correlating with the morphological stages. The initial pH of 12.41 is characteristic of a saturated Ca(OH)_2_ solution. The concurrent low conductivity (~8.71 ms/cm) reflects the limited concentration of free ions due to the low solubility of Ca(OH)_2_. As the reaction proceeds (0–8 min), both parameters increase steadily. The pH rises to a maximum of 12.62, indicating a continuous net release of OH^−^ ions into the solution. This can be attributed to the dissolution of solid Ca(OH)_2_ to replenish Ca^2+^ consumed by carbonate precipitation, a process driven by the much lower solubility of CaCO_3_. The conductivity increases sharply to a peak of 15.54 ms/cm, resulting from the combined effect of the additional OH^−^ from Ca(OH)_2_ dissolution and the continuous introduction of Na^+^ and CO_3_^2−^ from the Na_2_CO_3_ solution. After the peak at ~8 min, a critical transition occurs: the conductivity exhibits a slight decrease. This signifies a shift in the ionic composition, specifically the depletion of the Ca^2+^ source. As Ca^2+^ (a divalent ion with high molar conductivity) is consumed and replaced by Na^+^ (a monovalent ion with lower molar conductivity), the overall conductivity drops, despite the high ionic strength.

In summary, the strong correlation between the solution chemistry and morphology provides insight into the formation and fracture mechanism of the NPCC chains. The period of increasing pH and conductivity (0–8 min) perfectly coincides with the phase of active chain assembly and growth observed in SEM. The rising OH^−^ concentration is critical, as it is known to adsorb preferentially onto specific crystal faces of CaCO_3_, inhibiting growth in those directions and promoting one-dimensional assembly via a “spatial steric effect” [33].

The peak in conductivity at ~8 min marks a turning point. The subsequent decrease signals the near-complete consumption of dissolved Ca^2+^ and the shift to Na^+^ as the dominant cation. The conductivity peaked at approximately 8 min and then gradually decreased, indicating an evolving ionic environment during the reaction. This trend may be associated with the consumption of dissolved Ca^2+^ and changes in the relative ionic composition of the solution. Such variations in ionic conditions could have influenced the stability of the transient chain-like assemblies observed in the SEM sequence.

**Figure 10:**
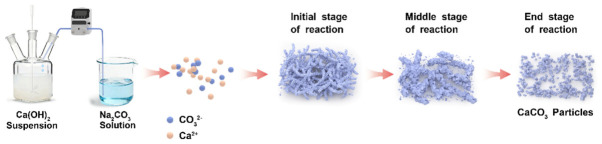
The schematic diagram of the formation mechanism of well-defined CaCO_3_.

In conclusion, the NPCC chain-like structure is a transient intermediate formed under specific ionic conditions. As shown in Figure 10, its assembly is driven by anisotropic growth suppression and particle attachment facilitated by high pH and ionic strength. Its eventual fracture is triggered by a shift in the ionic environment, primarily the depletion of Ca^2+^ and the consequent rise in electrostatic repulsion, leading to the final product of dispersed calcite nanoparticles.

## 4. Conclusions

In this study, nanoscale precipitated calcium carbonate (NPCC) with uniform morphology, good dispersibility, and smooth surfaces was successfully synthesized via a facile and additive-free route based on the precisely regulated reaction between Na_2_CO_3_ solution and Ca(OH)_2_ suspension. Through a systematic investigation of the crystallization process, this work provides insight into the formation and subsequent disintegration behavior of transient chain-like CaCO_3_ superstructures. These findings not only advance the current understanding of non-classical growth behavior in CaCO_3_ crystallization but also establish a simple, rapid, and economically viable strategy for the production of high-quality NPCC. The results offer significant guidance for the tailored regulation of CaCO_3_ morphology and particle size, with direct relevance to industrial mineralization processes and the rational design of advanced functional materials.

## Figures and Tables

**Table 1:** Variations in CaCO_3_ grain size at various aging times.

Aging Time (h)	Crystallite Size (nm)
0.5 h	78.7
1 h	79.0
2 h	73.3
4 h	63.8
8 h	49.6

## Data Availability

The original contributions presented in this study are included in the article. Further inquiries can be directed to the corresponding authors.

## Abbreviations

The following abbreviations are used in this manuscript:

NPCC	Nanoscale precipitated calcium carbonate
